# Hypertensive disorders in pregnancy and stillbirth rates: a facility-based study in China

**DOI:** 10.2471/BLT.18.208447

**Published:** 2018-06-12

**Authors:** Tao Xiong, Yi Mu, Juan Liang, Jun Zhu, Xiaohong Li, Jinke Li, Zheng Liu, Yi Qu, Yanping Wang, Dezhi Mu

**Affiliations:** a Department of Paediatrics, Key Laboratory of Birth Defects and Related Diseases of Women and Children of the Ministry of Education, West China Second University Hospital, Sichuan University, Chengdu, Sichuan, China.; b National Office for Maternal and Child Health Surveillance Sichuan University, Chengdu, China.

## Abstract

**Objective:**

To assess the association between hypertensive disorders in pregnancy and the stillbirth rate.

**Methods:**

We obtained all data from China’s National Maternal Near Miss Surveillance System for 2012 to 2016. Associations between hypertensive disorders in pregnancy and stillbirths, stratified by fetus number and gestational age, were assessed using Poisson regression analysis with a robust variance estimator.

**Findings:**

For the period, 6 970 032 births, including 66 494 stillbirths, were reported to the surveillance system. The weighted stillbirth rate in women with a hypertensive disorder in pregnancy was 21.9 per 1000 births. The risk was higher in those who had received few antenatal care visits or who were poorly educated. For singleton pregnancies, the adjusted risk ratio (aRR) for a stillbirth among women with hypertensive disorders in pregnancy compared with normotensive women was 3.1 (95% confidence interval, CI: 2.85–3.37). The aRR for hypertensive disorder subtypes was: 6.66 (95% CI: 5.57–7.96) for superimposed preeclampsia; 4.15 (95% CI: 3.81–4.52) for preeclampsia or eclampsia; 2.32 (95% CI: 1.87–2.88) for chronic hypertension; and 1.21 (95% CI: 1.08–1.36) for gestational hypertension. For multiple pregnancies, the association between stillbirths and hypertensive disorders in pregnancy was not significant, except for superimposed preeclampsia (aRR: 1.95; 95% CI: 1.28–2.97).

**Conclusion:**

To minimize the incidence of stillbirths, more attention should be paid to chronic hypertension and superimposed preeclampsia in singleton pregnancies and to superimposed preeclampsia in multiple pregnancies. Better quality antenatal care and improved guidelines are needed in China.

## Résumé

**Objectif:**

Évaluer l'association entre les troubles hypertensifs pendant la grossesse et le taux de mortinatalité.

**Méthodes:**

Toutes les données provenaient du Système de surveillance national des décès maternels évités de justesse de la Chine pour la période allant de 2012 à 2016. Les associations entre troubles hypertensifs pendant la grossesse et mortinaissances, stratifiées suivant le nombre de fœtus et l'âge gestationnel, ont été évaluées à l'aide d'une analyse de régression de Poisson et d'un estimateur de variance fiable.

**Résultats:**

Pendant la période considérée, 6 970 032 naissances, dont 66 494 mortinaissances, ont été enregistrées dans le système de surveillance. Le taux de mortinatalité pondéré chez les femmes présentant un trouble hypertensif pendant la grossesse était de 21,9 pour 1000 naissances. Le risque était plus élevé chez celles qui avaient bénéficié de peu de consultations prénatales ou qui avaient un faible niveau d'instruction. Pour les grossesses monofœtales, le risque relatif ajusté (RRa) de mortinaissance chez les femmes présentant des troubles hypertensifs pendant la grossesse comparées aux femmes normotendues était de 3,1 (intervalle de confiance de 95%, IC: 2,85–3,37). Le RRa des sous-types de troubles hypertensifs était de: 6,66 (IC 95%: 5,57–7,96) pour la pré-éclampsie surajoutée; 4,15 (IC 95%: 3,81–4,52) pour la pré-éclampsie ou l'éclampsie; 2,32 (IC 95%: 1,87–2,88) pour l'hypertension chronique; 1,21 (IC 95%: 1,08–1,36) pour l'hypertension gestationnelle. Pour les grossesses multiples, l'association entre mortinaissances et troubles hypertensifs pendant la grossesse n'était pas significative, hormis pour la pré-éclampsie surajouté (RRa: 1,95; IC 95%: 1,28–2,97).

**Conclusion:**

Afin de réduire l'incidence des mortinaissances, il faudrait accorder une plus grande attention à l'hypertension chronique et à la pré-éclampsie surajoutée lors des grossesses monofœtales ainsi qu'à la pré-éclampsie surajoutée lors des grossesses multiples. Des soins prénatals de meilleure qualité ainsi qu'une amélioration des directives sont nécessaires en Chine.

## Resumen

**Objetivo:**

Evaluar la asociación entre los trastornos hipertensivos en el embarazo y la tasa de mortinatos.

**Métodos:**

Todos los datos se obtuvieron del Sistema Nacional de Vigilancia de Casi Accidentes Maternos de China (National Maternal Near Miss Surveillance System) de 2012 a 2016. Las asociaciones entre los trastornos hipertensivos en el embarazo y los mortinatos, estratificadas según el número de fetos y la edad gestacional, se evaluaron según el análisis de regresión de Poisson con un estimador de varianza sólido.

**Resultados:**

Durante el periodo, se registraron en el sistema de vigilancia 6 970 032 nacimientos, incluidos 66 494 mortinatos. La tasa ponderada de mortinatos en mujeres con un trastorno hipertensivo durante el embarazo fue de 21,9 por cada 1000 nacimientos. El riesgo era mayor en aquellas que habían recibido pocas visitas de atención prenatal o tenían una educación deficiente. Para los embarazos sencillos, el coeficiente de riesgo ajustado (aRR) para un mortinato entre las mujeres con trastornos hipertensivos durante el embarazo en comparación con las mujeres normotensas fue de 3,1 (intervalo de confianza del 95%, IC: 2,85–3,37). El aRR para los subtipos de trastorno hipertensivo fue: 6,66 (IC del 95%: 5,57-7,96) para la preeclampsia superpuesta; 4,15 (IC del 95%: 3,81-4,52) para la preeclampsia o eclampsia; 2,32 (IC del 95%: 1,87-2,88) para la hipertensión crónica; 1,21 (IC del 95%: 1,08–1,36) para la hipertensión gestacional. Para los embarazos múltiples, la asociación entre mortinatos y trastornos hipertensivos en el embarazo no fue significativa, excepto en lo relativo a la preeclampsia superpuesta (aRR: 1,95; IC del 95%: 1,28-2,97).

**Conclusión:**

Para reducir al mínimo la incidencia de mortinatos, se debe prestar más atención a la hipertensión crónica y a la preeclampsia superpuesta en los embarazos sencillos y a la preeclampsia superpuesta en los embarazos múltiples. En China es necesaria una mejor calidad de atención prenatal y mejores directrices.

## ملخص

**الهدف:**

تقييم العلاقة بين اضطرابات ارتفاع ضغط الدم في الحمل ومعدل وفيات الرُضّع:

**الطريقة:**

حصلنا على جميع البيانات من النظام الوطني لمراقبة حالات نجاة الأمهات من الوفاة المؤكدة في الصين خلال الفترة من عام 2012 حتى عام 2016. تم تقييم حالات الارتباط بين اضطرابات ارتفاع ضغط الدم في الحمل ووفيات الرُضّع، مصنفة على حسب عدد الأجنة وعمر الحمل، باستخدام تحليل التحوف لبواسون، مع مُقدّر قوي للتباين.

**النتائج:**

خلال هذه الفترة، تم إبلاغ نظام المراقبة بـ 6970032 ولادة، بما في ذلك 66494 حالة وفاة للرُضّع. كان معدل وفيات الرُضّع المرجح عند النساء المصابات باضطراب ارتفاع ضغط الدم أثناء الحمل 21.9 لكل 1000 مولود. كان الخطر أعلى في هؤلاء السيدات اللاتي تلقين القليل من زيارات الرعاية السابقة للولادة، أو اللاتي كان حظهن من التعليم محدود. بالنسبة لحالات الحمل المنفردة، كانت نسبة المخاطر المعدلة (aRR) لوفيات الرُضّع بين النساء اللواتي تعانين من اضطرابات ارتفاع ضغط الدم في الحمل، مقارنة بالنساء اللاتي حظين بضغط دم معتدل، هي 3.1 (فاصل الثقة 95٪، 2.85 إلى 3.37). كانت نسبة المخاطر المعدلة لأنواع فرعية من اضطراب ارتفاع ضغط الدم: 6.66 (فاصل الثقة 95%: 5.57 إلى 7.96) لتسمم الحمل المتراكب؛ 4.15 (فاصل الثقة 95‏%: 3.81 إلى 4.52) لتسمم الحمل المتراكب أو الارتعاج؛ 2.32 (فاصل الثقة 95‏%: 1.87 إلى 2.88) لارتفاع ضغط الدم المزمن؛ 1.21 (فاصل الثقة 95‏%: 1.08 إلى 1.36) لارتفاع ضغط الدم أثناء الحمل. بالنسبة لحالات الحمل المتعدد، لم يكن الارتباط بين حالات وفيات الرُضّع واضطرابات ارتفاع ضغط الدم في الحمل كبيراً، باستثناء حالات تسمم الحمل المتراكمة (نسبة المخاطر المعدلة: 1.95؛ فاصل الثقة 95٪: 1.28-2.97).

**الاستنتاج:**

للتقليل من حالات وفيات الرّضع، ينبغي توجيه المزيد من الاهتمام لارتفاع ضغط الدم المزمن والتسمم المتراكب في الحمل المفرد، والتسمم المتراكب في حالات الحمل المتعددة. هناك حاجة إلى رعاية أفضل قبل الولادة، وكذلك مبادئ توجيهية محسنة في الصين. ****

## 摘要

**目的:**

旨在评估妊娠期高血压疾病与死胎率之间的联系。

**方法:**

我们从中国危重孕产妇监测系统中获取所需的 2012 至 2016 年间的所有数据。使用带有稳定方差估计值的 Poisson 回归系统对妊娠期高血压疾病与死胎率之间的关系进行了评估。死胎率按照胎儿数量和胎龄进行分层。

**结果:**

该期间内，上报至监测系统中的 6970032 例出生案例中，有 66494 例死胎案例。患有妊娠期高血压疾病的孕妇加权死胎率为每 1000 例中 21.9 例。几乎不做产前检查或受教育程度低的孕产妇风险更高。对于单胎妊娠，患有妊娠期高血压疾病的孕产妇与血压正常的孕产妇之间的调整风险率 (aRR) 为 3.1（95% 的置信区间，CI：2.85-3.37）。妊娠期高血压疾病子类型的调整风险率 (aRR) 为：慢性高血压并发子痫前期 6.66 (95% CI：5.57–7.96)；子痫前期子痫 4.15 (95% CI：3.81-4.52)；妊娠合并慢性高血压 2.32 (95% CI：1.87-2.88)；以及妊娠期高血压 1.21 (95% CI：1.08–1.36)。对于多胞胎，除了慢性高血压并发子痫前期 (aRR：1.95；95% CI：1.28–2.97)，死胎率与妊娠期高血压疾病之间的关系并不明显。

**结论:**

为最大程度降低死胎率，应对患有妊娠合并慢性高血压和慢性高血压并发子痫前期的单胎孕妇，以及患有慢性高血压并发子痫前期的多胞胎孕妇给予更多关注。中国需提高产前检查质量，完善孕期指导。****

## Резюме

**Цель:**

Оценить взаимосвязь между гипертензивными нарушениями во время беременности и уровнем мертворождаемости.

**Методы:**

Авторы получили все данные Национальной системы эпиднадзора за критическими состояниями беременных женщин в Китае за 2012–2016 годы. Взаимосвязь между гипертензивными нарушениями во время беременности и показателем мертворождаемости, стратифицированная по количеству плодов и гестационному возрасту, оценивалась с использованием регрессионного анализа Пуассона с устойчивой оценкой дисперсии.

**Результаты:**

За отчетный период в системе эпиднадзора имеются данные о 6 970 032 родах, в том числе 66 494 случая мертворождения. Взвешенный уровень мертворождаемости у женщин с гипертензивными нарушениями во время беременности составил 21,9 случая на 1000 родов. Более высокий риск был у женщин, нерегулярно посещавших врача с целью дородового наблюдения или с низким уровнем образования. Для одноплодной беременности показатель скорректированного отношения рисков (сОР) для мертворождения среди женщин с гипертензивными нарушениями во время беременности по сравнению с женщинами с нормальным артериальным давлением составил 3,1 (95%-й ДИ: 2,85–3,37). Показатель сОР для подтипов гипертензивных нарушений составил: 6,66 (95%-й ДИ: 5,57–7,96) для сочетанной преэклампсии; 4,15 (95%-й ДИ: 3,81-4,52) для преэклампсии или эклампсии; 2,32 (95%-й ДИ: 1,87–2,88) для хронической гипертензии; 1,21 (95%-й ДИ: 1,08–1,36) для гипертензии беременных. Для многоплодной беременности взаимосвязь между мертворождаемостью и гипертензивными нарушениями во время беременности была незначительной, за исключением сочетанной преэклампсии (сОР: 1,95; 95%-й ДИ: 1,28–2,97).

**Вывод:**

Для минимизации уровня мертворождаемости следует уделять больше внимания хронической гипертензии и сочетанной преэклампсии при одноплодной беременности, а также сочетанной преэклампсии при многоплодной беременности. В Китае требуется повысить качество дородового наблюдения и усовершенствовать нормативную документацию.****

## Introduction

Stillbirths constitute an important worldwide problem that has generally received little attention.^1^ There are an estimated 2.6 million stillbirths each year, with 98% occurring in low- and middle-income countries.^1^ In China, a rate of 8.8 per 1000 births was reported in 2016.^2^ Even in high-income countries, stillbirth remains a major, and potentially avoidable, health burden.^3^ As a high proportion are unexplained, better understanding could help reduce avoidable stillbirths and decrease perinatal mortality.^3^ Systematic efforts have been made to identify the causes. For example, it is known that stillbirths are closely associated with pregnancy complications and that hypertensive disorders in pregnancy are the most common pregnancy complications.^4^ Such disorders, which include chronic hypertension, superimposed preeclampsia, preeclampsia–eclampsia and gestational hypertension, occur in 3 to 8% of pregnancies worldwide.^5^^,^^6^ These four subtypes may have different pathological mechanisms and clinical manifestations and may, therefore, play different roles in stillbirth.^7^

Few large-scale studies have investigated the relationship between the different hypertensive disorders in pregnancy and stillbirth. Moreover, little is known about whether the number of fetuses modifies their effect on the risk. Most previous studies of women with hypertensive disorders in pregnancy have been limited to singleton pregnancies and have shown that they are associated with an increased incidence of stillbirth.^5^^,^^7^^–^^12^ In contrast, hypertensive disorders appear to have a beneficial effect on fetal survival in twin pregnancies.^13^ An exploration of the different effects of hypertensive disorders in pregnancy on the risk of a stillbirth in singleton and multiple pregnancies would help improve patient management and prevent fetal deaths.

For this study, we hypothesized that each subtype of hypertensive disorders in pregnancy influences the risk of stillbirth in a different way and that the influence varies between singleton and multiple pregnancies. Our specific aims were to investigate the association between these disorders and their subtypes and the stillbirth rate and to determine how that association varies with fetus number and gestational age, with the goal of improving clinical practice in China.

## Methods

We obtained data on pregnancies and pregnancy outcomes from China’s National Maternal Near Miss Surveillance System for 1 January 2012 to 31 December 2016. The surveillance system was established in October 2010 and covers 441 member hospitals, each of which manages more than 1000 deliveries annually. The hospitals are located in 326 districts or counties throughout 30 provinces in mainland China, excluding Tibet. Since certain districts and counties did not have hospitals with the minimum required number of births, especially in rural areas, large hospitals in urban districts were oversampled, particularly in central and western regions.^2^

We restricted our analysis to births that occurred after 28 or more weeks’ gestation or where the birthweight was 1000 g or more, in accordance with the World Health Organization’s (WHO’s) definition of a third-trimester stillbirth.^1^ However, in several previous studies of hypertensive disorders in pregnancy, the definition of a fetal death was a death at or after either 20 or 24 weeks. The reasons for this difference are that the perinatal period is defined as starting at 28 weeks in China and our adoption of WHO’s definition of a stillbirth.

In China, gestational age is generally estimated from the time of the last menstrual period or, when the date of the last period is unknown, on the basis of ultrasound findings. In this study, we expressed gestational age-specific stillbirth rates as stillbirths per 1000 births. Diagnostic criteria for hypertensive disorders in pregnancy vary between guidelines. We divided hypertensive disorders reported in the National Maternal Near Miss Surveillance System into four categories according to American College of Obstetricians and Gynecologists’ guidelines:^14^ (i) chronic hypertension; (ii) superimposed preeclampsia; (iii) preeclampsia or eclampsia; and (iv) gestational hypertension. Chronic hypertension was defined as hypertension (i.e. a systolic blood pressure of 140 mmHg or higher or a diastolic pressure of 90 mmHg or higher) before pregnancy or before 20 weeks’ gestation. Superimposed preeclampsia was defined as chronic hypertension associated with preeclampsia. Preeclampsia was defined as hypertension and proteinuria after 20 weeks’ gestation or hypertension plus the involvement of at least one organ or system. Eclampsia was diagnosed when preeclampsia progressed to the convulsive phase. Gestational hypertension was defined as new-onset hypertension that occurred after 20 weeks’ gestation with blood pressure normalization by 12 weeks postpartum.

We classified China’s regions as eastern, central or western according to standard definitions and divided hospitals into three levels (i.e. levels 1 to 3, where level 1 represents the smallest hospitals and level 3 the largest) according to: (i) the number of beds; (ii) the types of clinical departments; (iii) the number of medical personnel; (iv) the type and quantity of medical equipment; and (v) hospital funding.^2^ We also categorized data on the number of antenatal care visits made by the woman, the mother’s educational level, marital status, age at delivery and parity, the delivery method and the fetus’ gender.

### Data analysis

We excluded three of the 441 hospitals because they did not report data after 2012. In some remote counties, a few women gave birth in township hospitals, which were not included in the National Maternal Near Miss Surveillance System. Consequently, women giving birth at hospitals included in the surveillance system may not have been exactly representative of the local population. To account for this, we weighted the proportion of stillbirths in the surveillance system’s sampling distribution of the population according to China’s 2010 census, as detailed in other publications.^2^^,^^15^

We calculated the overall stillbirth rate in women with and without a hypertensive disorder in pregnancy and the rate in women with the four different types of hypertensive disorder, stratified by gestational age at birth. In addition, we estimated the stillbirth rate for singleton and multiple births among women with different hypertensive disorders. To identify the possible association between gestational age and the stillbirth rate, we categorized the births as occurring at a gestational age of: (i) less than 28 weeks (several babies with a birth weight of 1000 g or more were born at a gestational age under 28 weeks); (ii) 28 to 31 weeks; (iii) 32 to 36 weeks; or (iv) 37 weeks or more. The stillbirth rate in normotensive pregnancies was used as a reference.

Normally, Poisson regression analysis is regarded as an appropriate approach to analysing the risk of rare events, such as stillbirths. However, it will overestimate the error in the estimated relative risk when stillbirths are recorded as binomial data. This can be overcome by employing a robust error variance procedure known as sandwich estimation.^16^ Therefore, we performed a Poisson regression analysis with a robust variance estimator to examine the association between the different subtypes of hypertensive disorder in pregnancy and the proportion of stillbirths.^17^ We calculated adjusted relative risks (aRRs) and 95% confidence intervals (CIs) after weighting for the sampling distribution of the population and adjusting for: (i) the clustering of births within hospitals; (ii) region; (iii) hospital level; (iv) antenatal care; (v) the mother’s educational level, marital status, age and parity; (vi) the delivery method; (vii) the fetus’ gender; and (viii) other factors thought to be associated with stillbirth. These other factors included: (i) a ruptured uterus; (ii) placenta praevia; (iii) abruptio placentae; (iv) unspecified antepartum haemorrhage; (v) heart disease; (vi) embolism or thrombophlebitis; (vii) hepatic disease; (viii) anaemia (i.e. a haemoglobin level less than 11 g/dL); (ix) renal disease, including urinary tract infection; (x) lung disease, including upper respiratory tract infection; (xi) human immunodeficiency virus infection and acquired immune deficiency syndrome; (xii) connective tissue disorders; (xiii) gestational diabetes mellitus; and (xiv) cancer. The most robust and stable model was identified by examining its multicollinearity and goodness of fit. Statistical analyses were performed used Stata v. 14.2 (StataCorp LP., College Station, United States of America). The National Maternal Near Miss Surveillance System was approved by the ethics committee of the West China Second University Hospital, Sichuan University, China (Protocol ID: 2012008; date of approval: 3 March 2012).

### Results

**Fig. 1:**
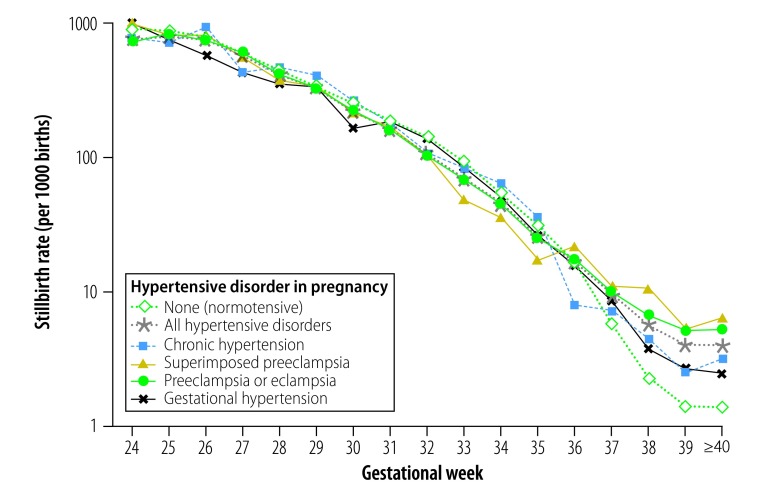
Stillbirth rate, by gestational week and hypertensive disorders in pregnancy, China, 2012–2016

Note: The stillbirth rate was weighted for the sampling distribution of the population covered by the Chinese National Maternal Near Miss Surveillance System.

Between 2012 and 2016, 66 494 stillbirths were recorded among 6 970 032 births in the National Maternal Near Miss Surveillance System. The weighted stillbirth rate was 21.9 per 1000 births in women with a hypertensive disorder in pregnancy and 8.4 per 1000 in normotensive women. The risk decreased as gestational age increased for all women and for all subtypes of hypertensive disorder (Fig. 1). Interestingly, at term (i.e. 37 weeks’ gestation or later) the rate was markedly higher in women with a hypertensive disorder, irrespective of subtype, than in normotensive women. The stillbirth rate was greater in women with a hypertensive disorder regardless of region, hospital level, antenatal care, the woman’s educational level, marital status, age or parity, the delivery method or the fetus’ gender. However, the rate was similar in hypertensive and normotensive women younger than 20 years.

Among women with a hypertensive disorder in pregnancy, the stillbirth rate was strongly influenced by sociodemographic characteristics (Table 1). For example, the rate was lower in east China than in other regions and lower in level-1 hospitals than in other levels. Moreover, a stillbirth was more likely if the woman had received few antenatal care visits, was poorly educated, was single, widowed or divorced, had a vaginal delivery, had high parity or was older than 40 years.

**Table 1:** Sociodemographic characteristics of women who had a stillbirth, by presence of hypertensive disorders in pregnancy, China, 2012–2016

Sociodemographic characteristic	Women with hypertensive disorders in pregnancy(*n* = 270 982)			Normotensive women(*n* = 6 699 050)	
	No. stillbirths	Weighted stillbirth rate,^a^ per 1000 births		No. stillbirths	Weighted stillbirth rate,^a^ per 1000 births
**Region of China**					
East	1 480	17.3		13 727	7.0
Central	2 953	24.2		23 772	8.2
West	2 333	24.4		22 229	10.1
**Hospital level^b^**					
Unknown	194	16.5		2 779	7.6
Level 1	106	9.4		2 727	6.0
Level 2	1 485	14.3		23 401	7.5
Level 3	4 981	32.0		30 821	10.7
**Antenatal care visits**					
None	467	68.5		4 676	42.5
1–3	1 532	51.8		15 623	28.0
4–6	2 488	25.0		21 715	8.6
7–9	1 112	13.1		9 552	4.4
≥ 10	625	8.1		5 161	2.6
Unknown	542	46.0		3 001	15.0
**Mother's education**					
None	172	51.9		1 058	27.3
Primary school	616	36.3		3 905	15.7
Middle school	2 173	21.2		22 423	8.8
High school	1 965	23.0		17 271	8.7
College or higher	1 522	16.2		13 669	5.9
Unknown	318	38.7		1 402	8.4
**Marital status**					
Single, widowed or divorced	172	37.1		3 387	31.8
Married	6 593	21.7		56 317	8.0
Unknown	1	12.5		24	14.8
**Mother's age, years**					
< 20	174	20.2		4 578	20.3
20–24	991	17.2		13 686	8.2
25–29	1 853	17.3		19 801	6.6
30–34	1 713	23.4		11 079	7.6
35–39	1 201	29.0		5 136	10.5
≥ 40	435	32.3		1 801	17.4
Unknown	399	45.6		3 647	15.3
**Delivery method**					
Vaginal	4 601	52.6		52 669	13.0
Caesarean section	2 165	10.2		7 059	2.2
**Fetus' gender**					
Female	3 177	20.6		26 255	7.8
Male	3 086	20.0		27 437	7.3
Unknown	503	513.8		6 036	516.7
**Parity^c^**					
Nulliparous	3 090	16.3		32 371	7.6
1	2 750	27.6		21 098	8.3
2	725	37.6		4 898	14.4
≥ 3	196	45.8		1 312	21.7
Unknown	5	25.7		49	17.5
Total	6 766	21.9		59 728	8.4

^a^ Weighted for the sampling distribution of the population covered by the Chinese National Maternal Near Miss Surveillance System.

^b^ We divided hospitals into three levels (where level 1 represents the smallest hospitals and level 3 the largest) according to was defined as (i) the number of beds; (ii) the types of clinical departments; (iii) the number of medical personnel; (iv) the type and quantity of medical equipment; and (v) hospital funding.

**^c^** Parity was based on the number of previous deliveries after 28 weeks’ gestation.

In singleton pregnancies, the risk of a stillbirth in women with a hypertensive disorder in pregnancy was significantly higher than in normotensive women (aRR: 3.1; 95% CI: 2.85–3.37; Table 2). For pregnant women with chronic hypertension, the aRR was 2.32 (95% CI: 1.87–2.88); for those with superimposed preeclampsia, it was 6.66 (95% CI: 5.57–7.96); for those with preeclampsia or eclampsia, it was 4.15 (95% CI: 3.81–4.52); and, for those with gestational hypertension, it was 1.21 (95% CI: 1.08–1.36). In addition, each hypertensive disorder subtype significantly increased the risk of stillbirth at all gestational ages, except the presence of chronic hypertension or gestational hypertension at less than 28 weeks’ gestation. In contrast, for multiple pregnancies, the association of hypertensive disorders was generally not significant (Table 3): only superimposed preeclampsia was associated with a significantly increased risk overall (aRR: 1.95; 95% CI: 1.28–2.97) and this increase was observed only at less than 28 weeks’ gestation (aRR: 2.85; 95% CI: 1.86–4.37) and at 28 to 31 weeks’ gestation (aRR: 2.53; 95% CI: 1.39–4.61).

**Table 2:** Association between hypertensive disorders in pregnancy and stillbirths for singleton births, China, 2012–2016

Gestational age and type of hypertensive disorder in pregnancy	No. stillbirths^a^	Weighted stillbirth rate,^a^ per 1000 births	Adjusted RR (95% CI)^a,b^
**< 28 weeks’ gestation**			
None (normotensive)	9 130	779.4	Reference
All hypertensive disorders in pregnancy	373	713.1	1.15 (1.08–1.22)
Chronic hypertension	38	691.0	1.06 (0.85–1.33)
Superimposed preeclampsia	49	733.6	1.26 (1.13–1.41)
Preeclampsia or eclampsia	252	717.3	1.16 (1.09–1.25)
Gestational hypertension	34	683.3	1.02 (0.82–1.25)
**28–31 weeks’ gestation**			
None (normotensive)	17 208	329.8	Reference
All hypertensive disorders in pregnancy	2 679	277.7	1.58 (1.48–1.67)
Chronic hypertension	133	333.9	1.56 (1.35–1.80)
Superimposed preeclampsia	281	272.8	1.74 (1.57–1.92)
Preeclampsia or eclampsia	2 075	273.4	1.60 (1.51–1.71)
Gestational hypertension	190	297.0	1.25 (1.09–1.43)
**32–36 weeks’ gestation**			
None (normotensive)	15 093	46.1	Reference
All hypertensive disorders in pregnancy	2 013	47.4	2.00 (1.86–2.15)
Chronic hypertension	93	44.9	1.70 (1.39–2.09)
Superimposed preeclampsia	113	44.4	2.28 (1.85–2.81)
Preeclampsia or eclampsia	1 559	48.4	2.26 (2.09–2.44)
Gestational hypertension	248	44.1	1.26 (1.09–1.45)
** ≥ 37 weeks’ gestation**			
None (normotensive)	11 115	1.8	Reference
All hypertensive disorders in pregnancy	996	5.2	3.06 (2.81–3.34)
Chronic hypertension	50	3.7	2.22 (1.61–3.07)
Superimposed preeclampsia	26	8.5	4.90 (3.26–7.34)
Preeclampsia or eclampsia	677	6.6	4.00 (3.58–4.46)
Gestational hypertension	243	3.4	1.94 (1.67–2.25)
**Total^c^**			
None (normotensive)	55 015	8.0	Reference
All hypertensive disorders in pregnancy	6 117	22.4	3.10 (2.85–3.37)
Chronic hypertension	322	18.6	2.32 (1.87–2.88)
Superimposed preeclampsia	482	66.4	6.66 (5.57–7.96)
Preeclampsia or eclampsia	4 594	28.9	4.15 (3.81–4.52)
Gestational hypertension	719	8.6	1.21 (1.08–1.36)

CI: confidence interval; RR: relative risk.

^a^ Weighted for the sampling distribution of the population covered by the Chinese National Maternal Near Miss Surveillance System.

^b^ Adjusted for: the clustering of births within hospitals; region; hospital level; antenatal care; the mother’s education, marital status, age and parity; the delivery method; the fetus’ gender; and other factors thought to be associated with stillbirth, such as a ruptured uterus, placenta praevia, abruptio placentae, unspecified antepartum haemorrhage, heart disease, embolism or thrombophlebitis, hepatic disease, anaemia (i.e. a haemoglobin level < 11 g/dL), renal disease (including urinary tract infection), lung disease (including upper respiratory tract infection), human immunodeficiency virus infection and acquired immune deficiency syndrome, connective tissue disorders, gestational diabetes mellitus and cancer.

^c^ Totals included stillbirths for which the gestational age was unknown.

**Table 3:** Association between hypertensive disorders in pregnancy and stillbirths for multiple births, China, 2012–2016

Gestational age and type of hypertensive disorder in pregnancy	No. stillbirths	Weighted stillbirth rate, per 1000 births^a^	Adjusted RR^a,b^
** < 28 weeks’ gestation**			
None (normotensive)	406	335.5	Reference
All hypertensive disorders in pregnancy	20	321.8	1.41 (0.92–2.16)
Chronic hypertension	2	268.8	1.21 (0.27–5.35)
Superimposed preeclampsia	2	1000.0	2.85 (1.86–4.37)
Preeclampsia or eclampsia	13	350.8	1.47 (0.87–2.48)
Gestational hypertension	3	190.8	1.00 (0.24–4.11)
**28–31 weeks’ gestation**			
None (normotensive)	1303	102.2	Reference
All hypertensive disorders in pregnancy	189	115.3	1.68 (1.39–2.04)
Chronic hypertension	5	79.8	1.33 (0.55–3.25)
Superimposed preeclampsia	11	174.1	2.53 (1.39–4.61)
Preeclampsia or eclampsia	152	120.3	1.83 (1.48–2.25)
Gestational hypertension	21	88.5	1.09 (0.62–1.91)
**32–36 weeks’ gestation**			
None (normotensive)	1863	20.3	Reference
All hypertensive disorders in pregnancy	322	16.0	0.94 (0.83–1.07)
Chronic hypertension	11	15.6	0.89 (0.44–1.81)
Superimposed preeclampsia	3	8.8	0.58 (0.18–1.87)
Preeclampsia or eclampsia	250	15.7	0.93 (0.81–1.07)
Gestational hypertension	58	18.4	1.04 (0.78–1.39)
** ≥ 37 weeks’ gestation**			
None (normotensive)	1020	9.7	Reference
All hypertensive disorders in pregnancy	111	8.6	0.99 (0.77–1.27)
Chronic hypertension	5	12.9	1.73 (0.69–4.31)
Superimposed preeclampsia	4	21.2	3.05 (0.99–9.43)
Preeclampsia or eclampsia	76	8.0	0.90 (0.66–1.21)
Gestational hypertension	26	9.1	1.09 (0.70–1.71)
**Total^c^**			
None (normotensive)	4713	21.9	Reference
All hypertensive disorders in pregnancy	649	17.8	1.07 (0.97–1.18)
Chronic hypertension	26	21.1	1.26 (0.79–2.02)
Superimposed preeclampsia	20	31.7	1.95 (1.28–2.97)
Preeclampsia or eclampsia	495	17.8	1.08 (0.97–1.21)
Gestational hypertension	108	16.4	0.94 (0.74–1.18)

CI: confidence interval; RR: relative risk

^a^ Weighted for the sampling distribution of the population covered by the Chinese National Maternal Near Miss Surveillance System.

^b^ Adjusted for: the clustering of births within hospitals; region; hospital level; antenatal care; the mother’s education, marital status, age and parity; the delivery method; the fetus’ gender; and other factors thought to be associated with stillbirth, such as a ruptured uterus, placenta praevia, abruptio placentae, unspecified antepartum haemorrhage, heart disease, embolism or thrombophlebitis, hepatic disease, anaemia (i.e. a haemoglobin level < 11 g/dL), renal disease (including urinary tract infection), lung disease (including upper respiratory tract infection), HIV/AIDS, connective tissue disorders, gestational diabetes mellitus and cancer.

^c^ Totals included stillbirths for which the gestational age was unknown.

## Discussion

In our study of nearly 7 million pregnancies in China, including single and multiple pregnancies, we found that the risk of stillbirth was increased in women with a hypertensive disorder in pregnancy. Moreover, the stillbirth rates for pregnant women in China with a hypertensive disorder in pregnancy, and with its subtypes, were clearly higher than the rates reported in developed countries,^5^^,^^7^ possibly because of differences in the level of medical care, in guidelines or in antenatal care.

In China, the most recent version of guidelines on the diagnosis and treatment of hypertensive disorders in pregnancy were developed by the Chinese Society of Obstetrics and Gynaecology in 2015.^18^ These are evidence-based guidelines that address the actual situation in the country and take into account American, Australian, British and Canadian guidelines.^14^^,^^19^^–^^21^ As in the American guidelines, Chinese guidelines place a strong emphasis on the management of preeclampsia and eclampsia. However, there are differences. In general, the Chinese guidelines are not as comprehensive as the American. For example, with regard to the timing of delivery, Chinese guidelines do not clearly recommend that pregnant women with mild gestational hypertension or preeclampsia without severe features should not give birth after 37 weeks’ gestation, which may delay delivery in these pregnancies. Since we found that the risk of a stillbirth increased with gestational age, a delay in delivery may increase the stillbirth rate in these women.

A small number of population studies have investigated the association between hypertensive disorders in pregnancy and stillbirth in singleton births in Norway^7^^,^^8^ and the United States of America.^5^^,^^13^ Similarly, only a few studies conducted in China have been published.^12^^,^^22^ One study of singleton births in the United States found that hypertensive disorders in pregnancy were associated with an increased risk of stillbirth. However, this study was limited because the diagnosis of a hypertensive disorder in pregnancy did not distinguish between the different subtypes.^5^ In Norway, the risk of stillbirth in singleton births was increased among pregnant women with preeclampsia (RR: 1.45)^8^ and among those with gestational hypertension (RR: 1.46) or chronic hypertension (RR: 2.12).^7^ Findings in these studies are consistent with the increased aRRs for stillbirth observed in our study for women with a hypertensive disorder in pregnancy who had a singleton pregnancy.

For multiple births, we found that the risk of stillbirth was not significantly different between women with a hypertensive disorder in pregnancy and normotensive women. Multiple births, thus, appear to differ from singleton births. Two possible reasons might explain this difference. First, the sample size of the multiple birth group may have been too small to detect a difference. Second, hypertensive disorders in pregnancy might exert a protective effect on multiple fetuses. Two studies conducted in the United States, which examined only twin pregnancies, reported that hypertensive disorders in pregnancy have a protective effect against perinatal death.^13^^,^^23^

One advantage of our study is that we assessed the risk conferred by chronic hypertension and superimposed preeclampsia separately. Both subtypes are associated with adverse outcomes, including a small-for-gestational-age fetus, preterm birth, fetal congenital malformation and cardiovascular disease in the mother.^24^^–^^27^ Many previous studies investigating stillbirths have combined chronic hypertension with gestational hypertension or grouped superimposed preeclampsia with preeclampsia, which undermines the usefulness of identifying chronic hypertension.^5^^,^^7^^,^^13^ In our study, the aRR for stillbirth in women with chronic hypertension who had a singleton pregnancy was 2.32, which is consistent with the adjusted odds ratio of 2.6 reported in a systematic review of high-income countries^28^ and 2.62 found in an observational study.^29^ Notably, in our study the aRR for chronic hypertension was higher than that for gestational hypertension, which was 1.21. Moreover, this was the case for all gestational age categories. In addition, our finding is comparable to that in a population study conducted in Norway: the RR for stillbirth with chronic hypertension was 2.12 versus 1.46 with gestational hypertension.^7^

A study in the United States of America found that women with chronic hypertension had a higher risk of developing preeclampsia than women in the general population (RR: 7.7; 95% CI: 5.7–10.1).^28^ Interestingly, we found that the risk of stillbirth in women with superimposed preeclampsia was greater than that in women with preeclampsia or eclampsia for singleton pregnancies: the aRR was 6.66 and 4.15 in the two groups, respectively. Furthermore, superimposed preeclampsia was the only hypertensive disorder that significantly increased the risk of stillbirth in women with a multiple pregnancy. These observations are consistent with other studies which found that superimposed preeclampsia resulted in worse outcomes than preeclampsia.^30^ Given that the outcomes of chronic hypertension are severe, our findings support the need to improve the management of pregnant women with the condition. Chronic hypertension must be prevented from developing into superimposed preeclampsia. Unfortunately, current Chinese guidelines lack a detailed management plan for chronic hypertension or superimposed preeclampsia. These limitations could increase the stillbirth rate in women with the two conditions. Consequently, future guidelines should focus on their management.

The aRR for stillbirth among women with gestational hypertension and a singleton pregnancy in our study (i.e. 1.21) was comparable to the adjusted odds ratio of 1.3 reported in a systematic review of high-income countries.^31^ However, preeclampsia is among the strongest maternal risk factors associated with stillbirth.^32^ In our study, the aRR among women with preeclampsia or eclampsia and a singleton pregnancy was 4.15, which is higher than the adjusted odds ratio of 1.6 for preeclampsia and 2.2 for eclampsia reported in the systematic review.^31^ This finding might reflect gaps in the management of preeclampsia and eclampsia between China and developed countries.

The present study has several advantages. First, it included one of the largest retrospective cohorts of women with hypertensive disorders in pregnancy reported in the literature. Therefore, sufficient data were available to stratify the risk of stillbirth by gestational age. Second, we had sufficient information to estimate the risk in singleton and multiple pregnancies separately; previous studies have generally estimated the risk in either singleton or multiple pregnancies. Third, data on the subtypes of hypertensive disorders in pregnancy were available for calculating the stillbirth rate and aRR for each subtype. Finally, the study had broad geographic coverage in China and used common protocols to collect data from the National Maternal Near Miss Surveillance System.

One major limitation of our retrospective study was the difficulty of differentiating intrauterine effects from residual confounders. However, we calculated the risk of stillbirth due to hypertensive disorders in pregnancy by adjusting for other factors. Consequently, the aRRs are likely to be realistic. Another limitation was the difference between the population covered by the National Maternal Near Miss Surveillance System, which is a hospital-based surveillance system that oversamples large referral hospitals in urban districts, and the whole Chinese maternal population. We attempted to correct for this oversampling by weighting the data to reflect differences between the population distribution in urban districts and rural counties in each region. However, we were not able to determine whether this weighting fully adjusted for the oversampling.^2^ Finally, time delays between the occurrence of a fetal death and recognition of the stillbirth may have increased the reported gestational age of stillbirths. However, this inaccuracy is likely to be limited because pregnant women with hypertensive disorders receive more frequent antenatal care, particularly at term.

Our findings might have important implications. For singleton pregnancies, hypertensive disorders increase the risk of stillbirth. Moreover, as the risk varies among the four subtypes of hypertensive disorders in pregnancy, different management strategies would be beneficial. Previous studies of these disorders mainly focused on specific risks to either fetuses or mothers. Future studies should consider the balance of benefits and risks to both fetus and mother and should include not only short-term outcomes, such as stillbirth and neonatal health, but also the long-term neural development of the child, cardiovascular risks for the mother after a hypertensive disorder in pregnancy and economic costs. In our study, a low maternal educational level and a low number of antenatal care visits were both associated with an increased risk of stillbirth in women with a hypertensive disorder in pregnancy. Therefore, the provision of regular, high-quality antenatal care and health education could help prevent stillbirths in these women. However, primary hospitals in China usually lack the experience to manage the risks associated with hypertensive disorders in pregnancy. The dissemination of guidelines on these disorders may also help decrease the risk of stillbirth in affected women.
